# Ethnicity as a risk factor for gambling disorder: a large-scale study linking data from the Norwegian patient registry with the Norwegian social insurance database

**DOI:** 10.1186/s40359-023-01391-0

**Published:** 2023-10-25

**Authors:** Sarah Helene Aarestad, Eilin Kristine Erevik, Otto Robert Frans Smith, Mark D. Griffiths, Tony Mathias Leino, Rune Aune Mentzoni, Ståle Pallesen

**Affiliations:** 1 Department of Psychosocial Science, University of Bergen, Bergen, Norway; 2 Norwegian Competence Center for Gambling and Gaming Research, University of Bergen, Bergen, Norway; 3 Department of Health Promotion, Norwegian Institute of Public Health, Bergen, Norway; 4 Department of Teacher Education, NLA University College, Bergen, Norway; 5 International Gaming Research Unit, Psychology Department, Nottingham Trent University, Nottingham, UK

**Keywords:** Gambling disorder, Ethnicity, Registry data

## Abstract

**Background:**

The study investigated ethnicity as a risk factor for gambling disorder (GD), controlling for demographics, citizenship, and years of residency in Norway.

**Methods:**

The sample comprised 65,771 individuals from a national patient registry (n = 35,607, age range 18–88 years) and a national social insurance database in Norway (n = 30,164, age rage 18–98 years). The data covered the period from 2008 to 2018.

**Results:**

The results showed that when controlling for age and sex, ethnic minorities were overall less likely than those born in Norway to be diagnosed with GD (odds ratio [OR] ranging from 0.293 to 0.698). After controlling for citizenship and years of residency in Norway, the results were reversed and indicated that ethnic minorities were overall more likely to be diagnosed with GD (OR ranging from 1.179 to 3.208).

**Conclusion:**

The results suggest that citizenship and years of residency are important variables to account for when assessing the relationship between ethnicity and being diagnosed with GD. Our results may be explained by people from ethnic minority groups being more likely to experience gambling problems but less likely to seek contact with healthcare services for gambling problems.

**Supplementary Information:**

The online version contains supplementary material available at 10.1186/s40359-023-01391-0.

## Introduction

Gambling is a popular activity across many cultures [1] and during the past few decades it has become highly accessible due to an increase in online gambling [2]. Although the majority of those who participate in gambling are recreational players who do not experience negative consequences, a small minority lose control and develop gambling-related problems, or even worse, gambling disorder (GD) [3–5]. GD is now considered a non-substance addiction and is included in both the fifth edition of the *Diagnostic and Statistical Manual of Mental Disorders* [DSM-5; 6] and the 11th revision of the *International Statistical Classification of Diseases and Related Health Problems* [ICD-11; 7]. GD is characterised by repeated and frequent episodes of gambling behaviours that have a detrimental effect on the individual’s life (e.g., occupational, martial, economical, and/or social life). The prevalence of GD as diagnosed with the DSM ranges from 0.1 to 1.6% depending on the country, year of survey, and the assessment methods that have been used [5, 8].

Several different risk factors for GD have been identified such as younger age, being male, and having lower socioeconomic status [8]. Another risk factor that has been identified in the literature is ethnicity [9, 10]. In countries such as Canada [e.g., 11], United States [e.g., 12, 13], Australia [e.g., 14], and Denmark [e.g., 15] it has been reported that ethnic minority groups have a higher prevalence of GD than the ethnic majority. A study by Alegria, Petry [16] reported that the prevalence of GD was higher among Native and Asian Americans (2.3%) and Blacks (2.2%) compared to Whites (1.2%). There are likely multiple reasons why GD is more prevalent among ethnic minorities. One is that in some cultures there is a higher acceptance for gambling. An example of this is the Chinese culture where gambling is regarded as a social leisure activity. Consequently, exposure to gambling is often higher among Chinese compared to other ethnic groups [17]. Another potential reason could be that some ethnic minority groups have lower income and socioeconomical status, as well as higher rates of unemployment, compared to the majority group, which are factors known to be associated with an increased risk of developing GD [17]. Even though GD is more prevalent among ethnic minorities, fewer individuals from ethnic minorities appear to seek healthcare services and treatment for GD [18, 19]. This could reflect cultural barriers which may in part be explained by mental illnesses being associated with a high degree of shame and stigma in some ethnic groups [20]. Poor acculturation could also affect help-seeking behaviour as there could be language barriers or a lack of information about healthcare services available [21]. Another potential reason for lack of treatment-seeking from professionals is that in some cultures it is more common to seek help from family or local community than seeking professional healthcare services [21].

However, it is worth noting that most of the previous studies examining ethnicity as a risk factor for GD have been carried out in the US which has a different composition of ethnic minority groups compared to Nordic countries like Norway. While the dominant ethnic minorities in the US population are Hispanic/Latino (18.7%), Black/African Americans (12.4%), and Asians (6.0%) [22], the ethnic minorities that are predominant in the Norwegian population are European (9.0%), Asian (6.3%), and African (2.7%) [23]. It is therefore of value to investigate the association between ethnicity and GD in a Norwegian context. Additionally, a major limitation with most previous research concerning the association between ethnicity and GD is that it is almost exclusively based on self-report data. Self-report is not as reliable in part due to factors such as social desirability, which could potentially be higher in ethnic minorities where GD is perceived as more shameful than in the majority culture [e.g., 20]. Registry could overcome such limitations. However, when considering that several studies have found that immigrants exhibit lower health seeking behaviour in general [e.g., 24, 25], not limited to GD [18, 19], it could be hypothesised that researchers might not find evidence for ethnic minorities being overrepresented when using official health registry data to examine GD treatment-seekers. Against this backdrop the present study investigated ethnicity as a risk factor for being diagnosed with GD in the Norwegian population using official health registry data, and if length of stay in Norway and citizenship, as indicators of immigrant integration, modified the relationship between ethnicity and GD.

## Method

### Sample and procedure

The sample in the present study comprised 65,771 individuals. The sample stemmed from two national databases, the Norwegian Patient Registry [NPR; 26] and the Norwegian Social Insurance Database [FD-trygd; 27, 28]. Data from the two databases were linked using unique National Identity numbers.

The NPR is owned and funded by the Norwegian Directorate of Health. Furthermore, the registry covers all public specialist health-care services in Norway, including private institutions and medical specialists contracted to the regional health authorities [26]. That is, those receiving treatment in institutions (or private therapies) that are not contracted by regional health authorities are not registered in NPR. The registry contains detailed health information, including medical diagnoses, and has complete data going from 2008 and onwards. This database provides data concerning all individuals in Norway over the age of 18 years that have received a GD diagnosis (F63.0) through the specialist healthcare services, as well as the time they received their diagnosis, their age and sex.

FD-trygd consist of data from the Norwegian Social Insurance database where social insurance benefits and payments are recorded. The database is complete for the whole Norwegian population. The data include information from administrative registries from the Norwegian Labour and Welfare administration (NAV), the former State Public Employment Service, and Statistics Norway. FD-trygd contains information regarding demographics, including age, sex, country of birth (which was used as a proxy for ethnicity), and citizenship, as well as work status and social benefits for the Norwegian population. The database was established in 2000, but it contains complete records of citizenship and country of birth since 1992 (citizenship) and October 1964 (country of birth), respectively. Any records prior to these dates have been added to the database when they have been available. Age, citizenship, and years of residency were measured with reference to 2018 in the analyses, while GD diagnosis was registered when first diagnosed between 2008 and 2018.

The GD group (*n* = 5131) was compared to a random sample of sex- and aged-matched individuals from FD-trygd (*n* = 30,164), representing the general Norwegian population, as well as a random sample of age- and sex-matched individuals suffering from any other disorders than GD from the NPR (*n* = 30,476). Both control groups comprised of approximately six times as many individuals as the GD group, and the GD group and the two control groups were compared in terms of country of birth.

### Statistical analyses

All statistical analyses were conducted with SPSS version 27. The analysis comprised two parts. The first part examined ethnicity as a risk factor for being diagnosed with GD by comparing the GD group and the NPR control group. In this regard, two binominal logistic regression analyses were conducted, where GD diagnosis (0 = No diagnosis, 1 = GD diagnosis) comprised the outcome variable, and ethnicity (assessed in terms of country of birth) comprised the exposure variable. In the first analysis, age (continuous variable) and sex (0 = Male, 1 = Female) were controlled for. The second analysis comprised a fully adjusted analysis where citizenship (categorical variable) and years of residency in Norway (continuous variable) were added as adjustment/confounding variables in addition to age and sex. For the second part, the same logistic regression analyses were repeated, but here the ethnicity of the individuals in the GD group was compared with the FD-trygd control group comprising individuals representing the general population.

## Results

The GD group comprised 81.8% men (*n =* 4,195) and 18.2% women (*n =* 936), with a mean age of 40.9 years (SD = 11.7; range 18–88 years). The NPR control group comprised 81.6% men (*n =* 24,870) and 18.4% women (*n =* 5,606), with a mean age of 41.0 years (SD = 11.7; range: 18–88 years). The FD-trygd control group comprised 81.4% men (*n =* 24,541) and 18.6% women (*n =* 5,623), with a mean age of 41.0 years (SD = 11.7; range: 18–98 years). Table 1 shows the descriptive statistics for the three groups.

**Table 1:** Descriptive statistics regarding country of birth and citizenship for the gambling disorder group and the two control groups: the Norwegian Patient Registry and the Norwegian Social Insurance Database (FD-trygd)

	Gambling disorder (*n =* 5131)		The Norwegian Patient Registry (*n =* 30,476)		The Norwegian Social Insurance Database (*n =* 30,164)	
	%	(*n*)	%	(*n*)	%	(*n*)
Country of birth						
Norway	82.6	(4237)	67.5	(20,582)	78.1	(23,545)
Asia	6.1	(311)	7.1	(2156)	5.5	(1666)
Europe excluding the Nordic Countries	5.4	(275)	14.9	(4532)	10.6	(3190)
Nordic countries	2.6	(135)	5.0	(1533)	2.0	(601)
Africa	2.4	(122)	3.2	(984)	2.5	(752)
North America	0.3	(13)	1.1	(344)	0.5	(146)
South and Central America	0.7	(36)	0.9	(278)	0.8	(227)
Oceania	0.0	(2)	0.2	(67)	0.1	(37)
Citizenship						
Norway	90.4	(4637)	71.8	(21,870)	82.0	(24,735)
Asia	2.3	(117)	4.7	(1443)	3.5	(1047)
Europe excluding the Nordic Countries	3.2	(163)	14.1	(4289)	9.8	(2948)
Nordic countries	2.8	(142)	5.1	(1568)	2.0	(600)
Africa	1.1	(54)	2.5	(748)	1.8	(558)
North America	0.1	(3)	1.0	(301)	0.3	(88)
South and Central America	0.2	(11)	0.5	(162)	0.4	(109)
Oceania	0.0	(2)	0.2	(59)	0.1	(27)

The results from the binominal regression analysis investigating ethnicity as a risk factor for being diagnosed with GD, when controlling for age and sex are presented in Table 2. The results indicated that among the individuals from the NPR control group there was a significantly lower likelihood of being diagnosed with GD if the individual was born in Asia, Europe, the Nordic countries, Africa, North America, South and Central America, and Oceania compared to being born in Norway. When comparing the individuals with GD to the control group stemming from FD-trygd, there was a statistically lower likelihood of being diagnosed with GD if the individual was born in Europe and North America compared to being born in Norway, while there was a statistically higher likelihood of being diagnosed if an individual was born in one of the other Nordic countries compared to being born in Norway.

**Table 2:** Logistic regression examining ethnicity as a risk factor for gambling disorder when controlling for age and sex. Here showing the Gambling Disorder group compared to the Norwegian Patient Registry control group and the Gambling Disorder group compared to the Norwegian Social Insurance Database (FD-trygd) control group

	The Norwegian Patient Registry (*n =* 35,607)						The Norwegian Social Insurance Database (*n =* 35,295)				
	*B*	*SE*	Wald	*OR*	*CI* for *OR* (95%)		B	SE	Wald	OR	CI for *OR* (95%)
Age	0.002	0.001	2.090	1.002	0.999–1.004		0.001	0.001	0.780	1.001	0.999–1.004
Sex											
Female	− 0.034	0.040	0.714	0.967	0.894–1.046		− 0.041	0.040	1.078	0.959	0.887–1.037
Country of birth^1^											
Asia	− 0.359	0.063	32.483	0.698***	0.617–0.790		0.036	0.064	0.309	1.036	0.914–1.175
Europe excluding the Nordic countries	− 1.227	0.064	362.892	0.293***	0.258–0.332		− 0.741	0.065	129.546	0.476***	0.419–0.541
Nordic countries	− 0.850	0.091	86.602	0.427***	0.357–0.511		0.221	0.097	5.220	1.247*	1.032–1.507
Africa	− 0.513	0.098	27.679	0.599***	0.495–0.725		− 0.109	0.099	1.219	0.896	0.738–1.089
North America	− 1.692	0.283	35.742	0.184***	0.106–0.321		− 0.703	0.290	5.872	0.495*	0.281–0.847
South and Central America	− 0.468	0.178	6.913	0.626**	0.442–0.888		− 0.125	0.180	0.484	0.882	0.620–1.256
Oceania	− 1.939	0.718	7.295	0.144**	0.035–0.588		− 1.210	0.726	2.776	0.298	0.072–1.238

*Note.* *p < .05; **p < .01; ***p < .001., *OR* = odds ratio, *CI* = 95% confidence interval, ^1^Norway comprised the reference group.

However, in the fully adjusted logistic regression analysis in which age, sex, citizenship, and years of residency in Norway comprised the adjustment/confounding variables, the results were mostly reversed. The results indicated that among the individuals from the NPR control group there was a significantly higher likelihood of being diagnosed with GD if they were born in Asia and the Nordic countries, while there was a statistically lower likelihood of being diagnosed if they were born in North America compared to being born in Norway (see Table 3). The covariate citizenship had a significant positive association with GD, meaning that individuals were more likely to be diagnosed with GD if they had Norwegian citizenship compared to if they had another citizenship.

**Table 3:** Logistic regression examining ethnicity as a risk factor for gambling disorder when controlling for age, sex, citizenship, and years of residency in Norway. Here showing the Gambling Disorder group compared to the Norwegian Patient Registry control group and the Gambling Disorder group compared to the Norwegian Social Insurance (FD-trygd) Database control group

	The Norwegian Patient Registry (*n =* 35,607)						The Norwegian Social Insurance Database (*n =* 35,295)				
	*B*	*SE*	Wald	*OR*	*CI* for OR (95%)		*B*	*SE*	Wald	*OR*	*CI* for OR (95%)
Age	0.000	0.002	0.001	1.000	0.995–1.004		0.008	0.003	10.034	1.008**	1.003–1.013
Sex											
Female	− 0.041	0.040	1.024	0.960	0.887–1.039		− 0.045	0.040	1.262	0.956	0.884–1.034
Citizenship											
Norwegian	1.604	0.080	405.340	4.973***	4.254–5.813		1.060	0.081	172.651	2.886***	2.464–3.380
Years of residency in Norway	− 0.004	0.002	2.862	0.996	0.992–1.001		0.006	0.002	6.887	1.006**	1.002–1.011
Country of birth^1^											
Asia	0.165	0.083	3.981	1.179*	1.003–1.387		0.545	0.087	39.401	1.724***	1.454–2.043
Europe excluding the Nordic countries	− 0.065	0.098	0.448	0.937	0.774–1.134		0.233	0.101	5.352	1.262*	1.036–1.537
Nordic countries	0.441	0.116	14.576	1.554***	1.239–1.949		1.166	0.122	90.988	3.208***	2.525–4.076
Africa	0.082	0.115	0.507	1.085	0.866–1.360		0.476	0.119	15.932	1.610***	1.274–2.034
North America	− 0.648	0.291	4.945	0.523*	0.295–0.926		− 0.187	0.296	0.398	0.830	0.464–1.482
South and Central America	0.160	0.189	0.719	1.174	0.811–1.699		0.359	0.190	3.579	1.432	0.987–2.078
Oceania	− 0.589	0.726	0.658	0.555	0.134–2.303		− 0.169	0.734	0.053	0.845	0.200–3.563

*Note*. *p < .05; **p < .01; ***p < .001, *OR* = odds ratio, *CI* = 95% confidence interval, ^1^Norway comprised the reference group.

When comparing the individuals with GD to the control group from FD-trygd in the fully adjusted analyses, there was a statistically higher likelihood of being diagnosed with GD if individuals were born in Asia, Europe, the Nordic countries, and Africa compared to if they were born in Norway (see Table 3). The covariates of age, citizenship (0 = not Norwegian, 1 = Norwegian), and years of residency in Norway all had significant positive associations with GD. Therefore, individuals who were older, had Norwegian citizenship, or had lived longer in Norway were more likely to be diagnosed with GD compared to individuals who were younger, had another citizenship, or had lived in Norway for a shorter duration.

## Discussion

The present study is the first worldwide to examine ethnicity as a risk factor for being diagnosed with GD using national official patient registry data. The data suggested that when adjusting for age and sex, those born in Norway were more likely than other ethnic groups to be diagnosed with GD when compared to the NPR control group, representing individuals with other illness diagnoses than GD, and the FD-trygd control group, representing the general population. However, after adjusting for citizenship and years of residency in Norway findings indicated that individuals with GD, when compared to age and gender adjusted controls in the NPR and the FD-trygd control groups, were more likely to be diagnosed with GD if they belonged to ethnic minority groups than individuals born in Norway. Consequently, results of the present study suggest that citizenship and years of residency in particular modified the relationship between ethnicity and being diagnosed with GD.

It was found that individuals in the NPR control group born in Norway were more likely to be diagnosed with GD than any of the other ethnic minority groups when adjusting for age and sex only. This stands in contrast to earlier findings where ethnic minority groups often appear to be overrepresented among individuals with GD [1, 12, 16, 29]. This finding may be explained by findings from previous research indicating that people from ethnic minority groups were less likely to seek contact with healthcare services for GD [18, 19]. This behaviour is not limited to gambling as several studies have shown that immigrants exhibit lower health seeking behaviour for other health conditions as well [24, 25]. There are several reasons for why this is the case, such as language barriers, acculturation stress, and lower awareness about available treatment options [19, 24, 30]. Other possible explanations could be cultural stigma which could be a potential barrier for seeking healthcare services because in some cultures mental health illnesses and problems such as gambling are associated with a high degree of shame and stigma, which could result in the individual avoiding seeking help from healthcare services [20]. In line with this perspective, one study examining helpline users in the USA showed that gamblers who were of an Asian-American background where 7.5 times more likely than individuals who were white to report suicide attempts [31], which could potentially suggest that this group may delay use of mental healthcare services until problems have escalated [21, 32].

A similar trend for the NPR control group was also found for the FD-trygd control group, where individuals born in Norway were more likely to be diagnosed with GD when compared to being born in Europe or North America. However, perhaps more surprisingly, those born in other Nordic countries were more likely to be diagnosed with GD than individuals born in Norway. This finding may reflect differences in gambling treatment availability across the Nordic countries or that those who emigrate to Norway from other Nordic countries overall may have characteristics (e.g., lower socioeconomic status) associated with higher risks of GD.

When controlling for age, sex, citizenship, and years of residency in Norway, the results for the NPR control group indicated that individuals born in Asia or the Nordic countries were more likely to be diagnosed with GD compared to individuals born in Norway. Individuals born in North America were less likely to be diagnosed with GD compared to individuals born in Norway. A possible explanation for this finding could be that there were very few individuals from North America included in the sample. Therefore, the findings could reflect random fluctuation or potentially selection effects. It is plausible that prevalence of GD among immigrants varies by country of birth and that potentially the group of North Americans who immigrates to Norway have high education, high socioeconomic status, and high salary jobs. Nonetheless, when fully adjusting for the covariates, the findings from the present study were more in line with previous research where ethnic minority groups often had a higher GD prevalence [e.g., 1] than the ethnic majority. When adjusting for citizenship we found that several of the non-Norwegian ethnic groups were more likely to be diagnosed than without this adjustment, which suggests that individuals who are more integrated, or potentially more assimilated, are individuals who more likely seek healthcare services for GD. The findings from the FD-trygd control group showed similar findings to the NPR control group with individuals born in Asia, Africa, Europe, and the Nordic countries being more likely to be diagnosed with GD than individuals born in Norway when controlling all the covariates. Additionally, the findings indicated that individuals who were older or had Norwegian citizenship or had lived in Norway for a longer period of time were more likely to be diagnosed with GD. When controlling for Norwegian citizenship and years of residency, again several of the non-Norwegian ethnic groups were more likely to be diagnosed than without this adjustment. This suggests that individuals from ethnic minority groups who are less integrated do not seek treatment for their gambling problems. This is in accordance with several studies that have reported that those belonging to ethnic minority groups do not seek treatment for their gambling problems [18–20]. In a study by Gainsbury et al. [19] only one-third of the individuals belonging to migrant and ethnic minority groups knew about services providing help related to gambling, specifically targeted to individuals from ethnic minority groups.

As a further argument for the main conclusion from this paper even in the control group from the NPR, consisting of patients from the Norwegian patient registry, individuals from ethnic minority groups were less likely to be diagnosed with GD, which could potentially suggest that they are less likely to seek treatment for GD than for other health problems. Therefore, the findings from the present study highlight that there is a need for more information regarding the treatment offers available for GD directed toward ethnic minorities and that steps should be taken to make relevant treatment more accessible for this group of individuals because they are often found to be overrepresented among individuals with GD. Thus, a major implication from our findings is the importance of adjusting for citizenship and years of residency when using registry data in understanding the relationship between ethnicity and being diagnosed with GD.

Overall, previous studies have shown that ethnic minorities have higher rates of problem gambling and GD than the ethnic majority [9]. This might reflect cultural differences in terms of how gambling is viewed [33]. Another reason may be that ethnic minorities often have low socioeconomic status, high levels of unemployment, and lower income, where gambling may be viewed as a way of improving the economic situation. Socioeconomical deprivation may also motivate gambling as a means of temporarily escaping stress and dysphoric feelings associated with being in a difficult situation [34]. Due to poor integration in the culture of the ethnic majority, shame and stigma, and barriers in terms of language and knowledge regarding health services, individuals from ethnic minorities typically underuse healthcare services [19]. This seems to be the case here. Therefore, efforts to lower the threshold for seeking treatment for GD (e.g., by adapting treatment in terms of language and culture) by ethnic minorities should be prioritized [35].

### Strengths and limitations

There are some limitations with using registry data because such data is not always a good proxy for real world problems. In the present study, ethnicity was categorized based on country of birth of the individuals. This is a common way of making a proxy for ethnicity based on registry data, although this has been debated in terms of ethical aspects, the very rough aspects of such proxies, and their lack of linking to self-definitions of ethnicity [36]. To more optimally assess ethnicity, a self-labelled question would have been more suitable. However, such data were not available in the registries used in the present study.

A potential limitation that is worth noting is that there were few individuals who were born in North America diagnosed with GD. Therefore, it is possible that the result for this group could be affected by random fluctuation, or it could potentially be explained by selection effects. It is plausible that prevalence of GD among immigrants varies by country of birth and potentially the group of North Americans who immigrates to Norway have higher socioeconomic status. It is also worth noting that there were 168 individuals that were born in Norway but did not have a Norwegian citizenship. Another potential limitation with the present study was that socioeconomic status was not controlled for. This could be of value by eliminating confounding variables because belonging to ethnic minority groups is often associated with lower socioeconomic status, which have also been identified as a risk factor for GD. Furthermore, far from all suffering from GD will receive a GD diagnosis. However, a strength with using registry data was the large sample size enabling adjustment for a number of different cofounding factors. Moreover, being able to use registry data from both the NPR and FD-trygd allowed comparison between individuals diagnosed with GD to two control groups, one random sample from the NPR and one random sample from FD-trygd which strengthens the conclusion validity of the present study. Another noticeable strength of the present study was the use of registry data, avoiding biases such as recall bias, social desirability bias, common method bias, etc., typically associated with self-report data. In addition, registry data are relatively complete, therefore the problem with low response rate, commonly experienced by survey data, is avoided. Hypothesised causal models were assumed, although the findings were based on observational data. Still, such models help differentiate between the interpretations of results associated with assumed exposure and confounding variables, respectively, avoiding the “Table 2 fallacy” [37]. Directed acyclic graphs of the models were made by DAGitty 3.0 and are available as supplementary material.

## Conclusion

The present study examined ethnicity as a risk factor for GD using national patient registry data and national social insurance registry data. The key findings suggested that it is plausible that individuals belonging to ethnic minority groups could be underrepresented among individuals seeking healthcare services for their gambling problems. However, when controlling for both citizenship and years of residency in Norway, the results were generally reversed, indicating that ethnic minority groups seem overall to be more likely to be diagnosed with GD. The adjusted findings suggest that individuals who are more assimilated or integrated into Norwegian society appear to be more likely to seek healthcare services for their gambling problems or potentially more assimilated, are the ones who more likely seek healthcare services for GD. However, when considering the findings from both the NRP control group and the FD-trygd control group it appears that those from ethnic minority groups are more reluctant to seek healthcare services for GD specifically compared to other health issues. Therefore, future studies should focus on treatment of GD for ethnic minority groups because these groups appear to be overrepresented among individuals with GD as well as underrepresented among groups less likely to seek help for their problems. Providing information regarding treatment of GD more readily available for ethnic minorities should therefore be a priority. The findings from the present study also highlights that there are some limitations to registry data and that citizenship and years of residency are important variables to adjust for when considering the relationship between ethnicity and GD diagnosis when using registry data.

### Electronic supplementary material

Below is the link to the electronic supplementary material.

Supplementary Material 1

## Data Availability

The datasets generated and/or analysed during the current study are not publicly available due to data provider agreement. Requests regarding the dataset can be sent to the corresponding author. Data are only available upon application to the data providers (the Norwegian Patient Registry and the Norwegian Social Insurance Database).
