# Correction: Ethnicity as a risk factor for gambling disorder: a large-scale study linking data from the Norwegian patient registry with the Norwegian social insurance database

**DOI:** 10.1186/s40359-023-01427-5

**Published:** 2023-11-06

**Authors:** Sarah Helene Aarestad, Eilin Kristine Erevik, Otto Robert Frans Smith, Mark D. Griffiths, Tony Mathias Leino, Rune Aune Mentzoni, Ståle Pallesen

**Affiliations:** 1 Department of Psychosocial Science, University of Bergen, Bergen, Norway; 2 Norwegian Competence Center for Gambling and Gaming Research, University of Bergen, Bergen, Norway; 3 Department of Health Promotion, Norwegian Institute of Public Health, Bergen, Norway; 4 Department of Teacher Education, NLA University College, Bergen, Norway; 5 International Gaming Research Unit, Psychology Department, Nottingham Trent University, Nottingham, UK

**BMC Psychology (2023) 11:355**

10.1186/s40359-023-01391-0

Following publication of the original article [1], it was brought to the attention of the journal that the following competing interests declaration for the fourth author, Mark D. Griffiths, had been omitted:

MDG has received research funding from *Norsk Tipping *(the gambling operator owned by the Norwegian government). MDG has received funding for a number of research projects in the area of gambling education for young people, social responsibility in gambling and gambling treatment from *Gamble Aware* (formerly the *Responsibility in Gambling Trust*), a charitable body which funds its research program based on donations from the gambling industry. MDG undertakes consultancy for various gambling companies in the area of player protection and social responsibility in gambling.

The declaration has since been added to the published article. The authors thank you for reading and apologize for any inconvenience caused.

