# Supplementary figures and images for: Ethnicity as a risk factor for gambling disorder: a large-scale study linking data from the Norwegian patient registry with the Norwegian social insurance database
Source: BMC Psychol. 2023 Oct 25;11:355. doi: 10.1186/s40359-023-01391-0 (PMC10601130; doi:10.1186/s40359-023-01391-0)

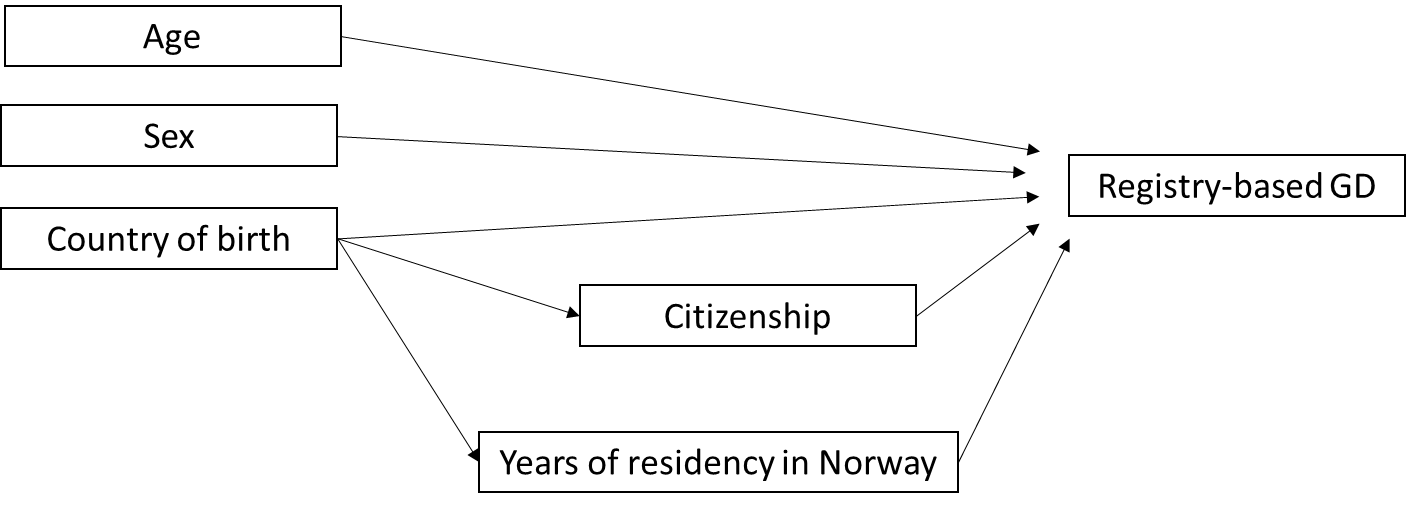


**Supplementary Figure 1.** *Assumed causal model in table 3.*

Supplement: Supplementary file 1 — Supplementary Material 1 [file 40359_2023_1391_MOESM1_ESM.docx]
